# Design and On-Orbit Validation of a Compact Wide-Swath Spaceborne SWIR Push-Broom Camera

**DOI:** 10.3390/s26082494

**Published:** 2026-04-17

**Authors:** Bo Cheng, Yongqian Zhu, Qianmin Liu, Jincai Wu, Bin Wu, Jiawei Lu, Zhihua Song, Bangjian Zhao, Chen Cao, Tianzhen Ma, Chunlai Li, Jianyu Wang

**Affiliations:** 1 Key Laboratory of Space Active Opto-Electronics Technology, Shanghai Institute of Technical Physics, Chinese Academy of Sciences (CAS), Shanghai 200083, China; chengbo21@mails.ucas.ac.cn (B.C.); zhuyongqian22@mails.ucas.ac.cn (Y.Z.); liuqianmin22@mails.ucas.ac.cn (Q.L.); jcwu@mail.sitp.ac.cn (J.W.); lujiawei@mail.sitp.ac.cn (J.L.); songzhihua@mail.sitp.ac.cn (Z.S.); matianzhen20@mails.ucas.ac.cn (T.M.); 2 University of Chinese Academy of Sciences, Beijing 100049, China; zhaobangjian@ucas.ac.cn (B.Z.); caochen@ucas.ac.cn (C.C.); 3 School of Physics and Optoelectronic Engineering, Hangzhou Institute for Advanced Study, University of Chinese Academy of Sciences, Hangzhou 310024, China; 4 College of Instrumentation & Electrical Engineering, Jilin University, Changchun 130061, China; wubin22@mails.jlu.edu.cn

**Keywords:** SWIR, InGaAs, through-focus MTF, remote sensing

## Abstract

**Highlights:**

**What are the main findings?**

- A compact dual-channel SWIR push-broom camera was designed and on-orbit validated, achieving a 187 km swath and 24 m nominal GSD with only 10.62 kg mass and ~12 W power consumption.
- An MTF-driven closed-loop focal plane leveling strategy using machinable SiC protrusions enabled confocal convergence across 16 mosaicked InGaAs detectors, yielding on-orbit LSF FWHM ≈ 1.38 pixels and stable SNR across multiple scenes.

**What are the implications of the main findings?**

- The results demonstrate a practical pathway to realize wide-swath, high-resolution SWIR payloads on resource-constrained platforms, significantly improving swath-to-mass efficiency relative to conventional systems.
- The proposed performance-driven focal plane compensation workflow (measurement–compensation–verification) is broadly applicable to compact multi-detector mosaicked imagers, reducing integration complexity without requiring per-detector mechanical refocusing.

**Abstract:**

To address the demand for wide-swath, high-resolution short-wave infrared (SWIR) imaging on resource-constrained spaceborne platforms, this study presents the design and on-orbit validation of a compact dual-channel push-broom (line-scanning) imaging system. The system adopts a transmissive optical architecture and a centralized, compact electronic control unit (ECU) configuration. By interleaving and mosaicking sixteen InGaAs linear array detectors, the system achieves an imaging swath of approximately 187 km and a nominal ground sampling distance of about 24 m, while maintaining a total instrument mass of 10.62 kg and a power consumption of approximately 12 W, thereby demonstrating a high level of integration and efficient resource utilization. To address focal plane consistency issues arising from multi-detector mosaicking, a closed-loop leveling method was developed using the modulation transfer function (MTF) as the primary performance metric. Through defocus estimation and quantitative correction of protrusions on a SiC substrate, convergence toward a unified confocal focal plane among multiple detectors was achieved. On-orbit image quality assessment indicates that the full width at half maximum (FWHM) of the line spread function (LSF) for both channels is approximately 1.38 pixels, with favorable signal-to-noise ratio (SNR) performance. These results validate the effectiveness of the proposed focal plane leveling strategy as well as the opto-mechanical-thermal design of the system. The proposed approach provides a practical pathway for the engineering implementation and consistency control of multi-detector mosaicked SWIR payloads under stringent resource constraints.

## 1. Introduction

Short-wave infrared (SWIR) remote sensing is of substantial value for coastal and inland water observations, particularly because it can provide auxiliary information for atmospheric correction in visible multispectral observations and enhance scene discrimination under complex atmospheric and aquatic backgrounds [1,2]. For such applications, both wide-area coverage and relatively high spatial resolution are often required simultaneously; accordingly, there is a clear demand for spaceborne SWIR imaging payloads with both wide swath and high resolution [3].

From a system design perspective, the ground sampling distance (GSD), swath width, and platform resources (e.g., mass, power consumption, and volume) are strongly coupled. Ultra-wide-swath operational payloads, such as Moderate Resolution Imaging Spectroradiometer (MODIS) and Visible Infrared Imaging Radiometer Suite (VIIRS), emphasize coverage efficiency but typically operate at spatial resolutions on the order of hundreds of meters [4,5]. In contrast, medium-to-high-resolution instruments such as Sentinel-2 Multispectral Instrument (MSI) and Landsat 8 Operational Land Image (OLI) achieve relatively large swath widths at spatial resolutions of approximately 20–30 m, but usually rely on platform resources with masses on the order of several hundred kilograms [6,7,8,9]. The system-level trade-off among swath width, spatial resolution, and platform resources becomes particularly pronounced for resource-constrained small satellites or constellation-based platforms [10,11,12,13,14].

From the perspective of imaging system development, the linear-array push-broom configuration has become a major technical route for spaceborne optical remote sensing at medium to high spatial resolution. Relevant studies on geometric modeling and engineering implementation can be traced back to the SPOT-1 era, indicating that the push-broom architecture has evolved into a relatively mature technological lineage [15]. Among representative payloads, EO-1/ALI adopted a push-broom architecture and demonstrated the feasibility of expanding the field of view by replicating modular detector units on the focal plane [16]. Subsequently, Landsat-8 OLI employed 14 focal plane modules (FPMs) to achieve a 185 km swath and 30 m-class multispectral observation [8,17], whereas Sentinel-2 MSI adopted 12 interleaved detector modules on both the visible and near-infrared (VNIR) and SWIR focal planes, enabling 10/20/60 m multispectral observations over a 290 km swath [9]. These payloads represent typical implementation pathways for wide-swath, medium- to high-resolution push-broom imaging systems and further demonstrate that expanding the effective focal-plane sampling capacity to simultaneously satisfy swath and resolution requirements is a well-established engineering approach.

For such systems, under given orbital altitude and target ground resolution constraints, increasing the swath fundamentally depends on enlarging the equivalent cross-track sampling number. Therefore, push-broom focal planes typically adopt a modular mosaicking configuration, combined with an interleaved arrangement along the flight direction to extend the effective sampling width and avoid gaps between adjacent strips [8,9,15,16,17]. However, while multi-detector mosaicking enhances swath capability, it also introduces new system-level challenges. One of the most critical among them is the inconsistency in imaging quality caused by focal plane height errors among detectors. Non-coplanarity of the focal plane directly affects the spatial resolution and uniformity of the entire image. In addition, the high-density arrangement of detectors further complicates electronic integration and thermal coupling, thereby making conventional alignment and integration procedures considerably more challenging.

In existing engineering practice, multi-detector mosaicking typically relies on three-dimensional metrology and alignment processes. However, such approaches fundamentally depend on structural dimensional tolerances and therefore represent a structure-driven integration paradigm. In highly integrated focal plane architectures characterized by compact layouts, strict space constraints, and limited capability for independent mechanical adjustment of individual detectors, traditional methods often become excessively complex or impractical. As a core metric describing the spatial frequency response of an optical imaging system, the MTF provides a sensitive indicator of image quality degradation caused by defocus [18,19,20,21]. Establishing a quantitative mapping between MTF and the equivalent defocus amount and incorporating it into the focal plane alignment procedure may therefore enable a transition from a structure-driven approach to a performance-driven integration paradigm.

To address these challenges, this study presents the design and on-orbit validation of a compact wide-swath, high-resolution SWIR push-broom camera system. The system employs sixteen indium gallium arsenide (InGaAs) linear array detectors arranged in an interleaved mosaic configuration, achieving an imaging swath of approximately 187 km and a nominal spatial resolution of 24 m. The complete system is integrated under strict resource constraints, with a total mass of 10.62 kg and a power consumption of approximately 12 W, resulting in a relatively high swath-to-mass ratio (see Table 1). The main contributions of this work are summarized as follows:

A compact ECU architecture is developed based on focal plane mosaicking, enabling high-density multi-channel signal integration and coordinated thermal-structural design.

A quantitative focal plane leveling method based on through-focus MTF analysis is proposed, establishing a performance-driven focal plane alignment framework. Through a unified process of measurement, fabrication, and re-evaluation, the method enables confocal optimization across multiple detectors.

Using on-orbit imaging data, system-level validation of spatial resolution and signal-to-noise ratio (SNR) is conducted, completing a closed-loop verification from ground testing to on-orbit performance assessment.

## 2. Materials and Methods

### 2.1. System-Level Design Constraints and Overall Architecture

For spaceborne push-broom wide-swath imaging systems operating at an orbital altitude H, the GSD can be approximated as:

(1) $$GSD=\frac{H\cdot p}{f},$$

where p denotes the pixel size and f represents the focal length of the optical system. The swath width of the imaging system can be expressed as:

(2) $$\mathrm{Swath}\;\approx \frac{N\cdot p\cdot H}{f},$$

where N is the total number of effective pixels along the cross-track direction.

Under a fixed orbital altitude and a predefined GSD requirement, the focal length f is largely determined by the spatial resolution constraint. Consequently, increasing the imaging swath primarily requires enlarging the effective number of pixels N. For a single linear detector, however, the pixel count is constrained by manufacturing processes and readout speed limitations, making it difficult to realize extremely long detector arrays while maintaining acceptable yield and noise performance. Therefore, for wide-swath imaging requirements, multi-detector mosaicking becomes a practical engineering solution.

Under resource-constrained platform conditions, the system design must balance three tightly coupled requirements: wide swath coverage on the order of hundreds of kilometers, spatial resolution at the level of several tens of meters, and lightweight/low-power implementation.

Based on these constraints, the proposed system employs sixteen indium gallium arsenide (InGaAs) linear array detectors arranged in an interleaved mosaicking configuration. By effectively increasing the number of pixels on the focal plane, the design simultaneously satisfies the swath and spatial resolution requirements. Compared with a simple side-by-side arrangement, the interleaved configuration provides higher packing efficiency in both the along-track and cross-track directions. On the one hand, for a given optical image-plane diameter, it improves the effective pixel coverage and reduces potential gaps between adjacent imaging strips. On the other hand, for the same target swath width, it allows a reduction in the image-plane size and primary optical aperture, thereby decreasing the mass of the optical system and the overall structural complexity.

Because the detectors are arranged in an interleaved staggered configuration, different detector rows observe the same ground scene at slightly different times during push-broom imaging. To account for this effect, the relative field-of-view relationship among detectors was first determined through ground geometric calibration, and the resulting geometric model was then combined with the spacecraft attitude and orbit information recorded in the image frame headers during on-orbit processing. In this way, the stagger-induced temporal offset is explicitly incorporated into subsequent geometric correction and inter-detector registration.

**Figure 1:**
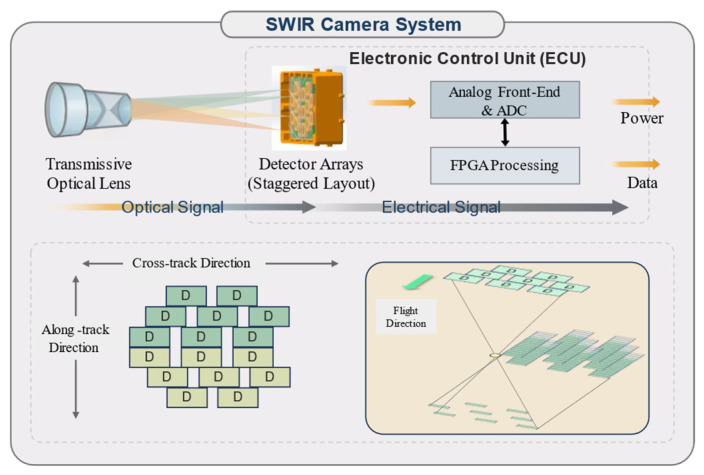
Composition and working mode of the short-wave infrared camera. “D” denotes a detector. The different colors represent the two spectral bands.

The overall architecture of the system is illustrated in Figure 1, consisting of a transmissive optical system and a highly integrated ECU. The optical subsystem is responsible for wide-swath imaging and image quality control, whereas the electronic subsystem performs multi-detector driving, data acquisition, and signal processing. Together, these subsystems form a lightweight and highly integrated SWIR push-broom imaging platform.

The principal imaging parameters and system specifications of the proposed instrument are summarized in Table 2.

### 2.2. Design of the Focal Plane Mosaicking Method

#### 2.2.1. Optimization of Circuit Design

**Figure 2:**
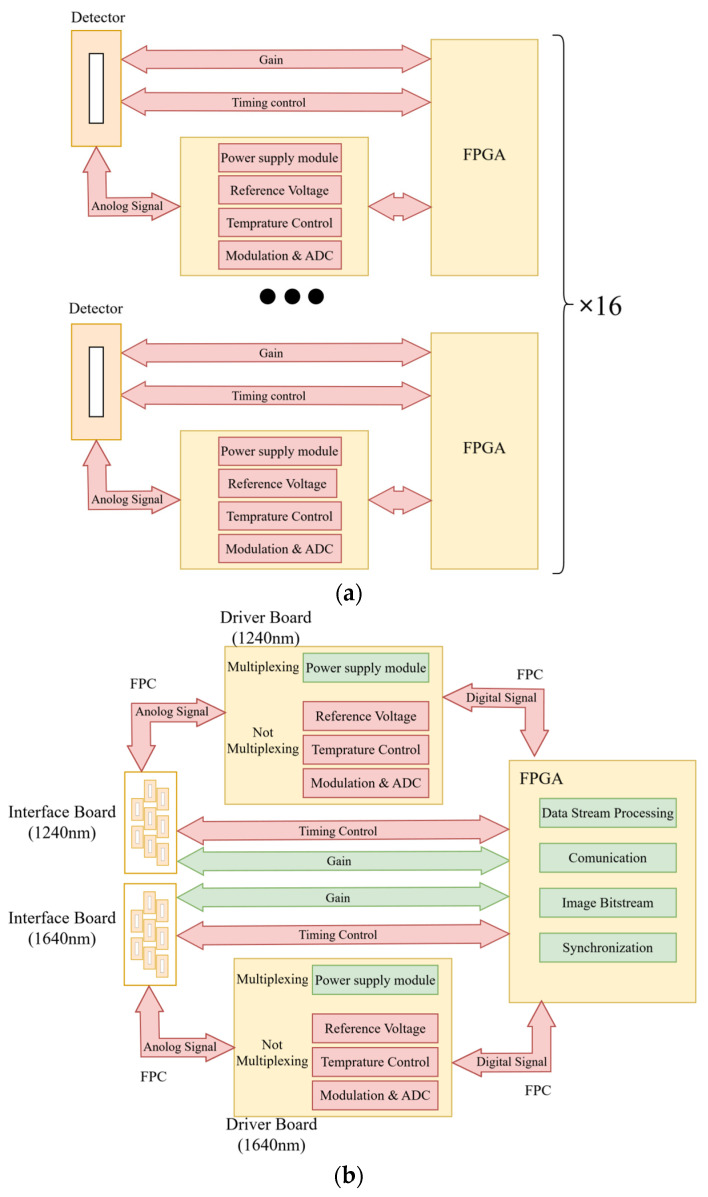
Comparison of circuit architectures for the multi-detector SWIR camera: (**a**) architecture based on replicated independent single-detector modules; (**b**) reconfigured and optimized centralized architecture.

The typical driving and readout circuit of a single indium gallium arsenide (InGaAs) linear array detector is illustrated in Figure 2a. The circuit mainly consists of a low-noise power supply module, a reference voltage configuration module, a high-precision timing control unit, an analog signal conditioning circuit, an analog-to-digital conversion module, and a temperature control unit. In addition, an independent FPGA core board is employed to implement logic control and data communication. Based on this architecture, a standardized single-detector module can be constructed.

However, in a multi-detector mosaicking system, a straightforward implementation based on the replication of a single module (×16) to expand the focal plane would introduce two major issues. First, each detector would require a complete set of driving and processing units, leading to a linear increase in electronic volume and power consumption, which is unfavorable for lightweight system design. Second, since each module operates relatively independently, stricter requirements would be imposed on structural installation and optical field-of-view alignment, significantly increasing the complexity of system integration.

To meet the requirements of hundred-kilometer-level swath coverage under strict constraints on mass and power consumption, the electronic system was reconstructed into a centralized architecture (Figure 2b). In the redesigned configuration, the circuitry is divided into several functional modules:

- Detector interface board, responsible for electrical connectivity and low-noise DC power supply;
- Driver board, performing analog signal conditioning, analog-to-digital conversion, and temperature control;
- FPGA core board, providing unified timing control, data processing, and external interface management for all sixteen detectors;
- Flexible printed circuit (FPC) boards, enabling high-density inter-board interconnections.

**Figure 3:**
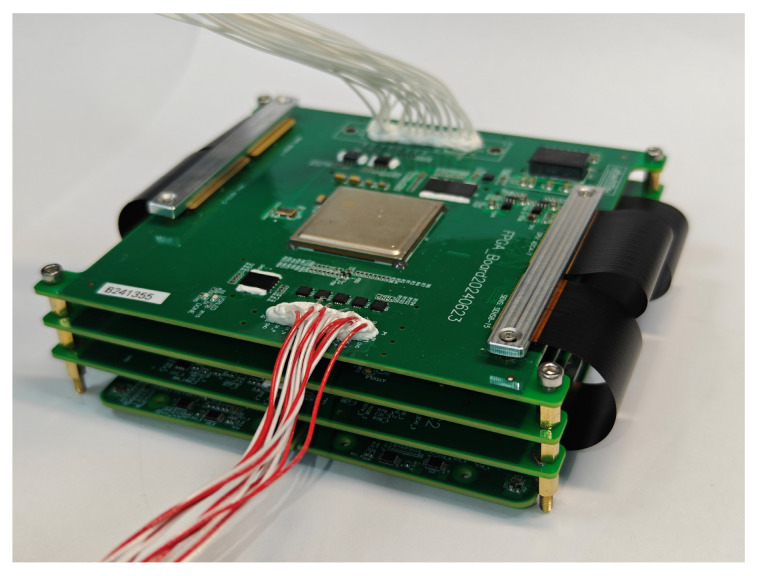
Assembled ECU.

Considering that approximately 520 inter-board signal connections are required, conventional board-to-board (BTB) connectors or daughterboard structures would significantly increase the occupied volume. Therefore, a hybrid solution combining compact BTB connectors with FPC interconnects was adopted to achieve high-density signal routing while optimizing the spatial layout. The assembled ECU is shown in Figure 3.

This centralized ECU architecture compresses the originally one-to-one detector–electronics configuration into a limited number of functional circuit boards. While maintaining signal integrity, it effectively reduces the electronic cost per imaging swath and constitutes a key system-level design strategy for achieving a lightweight and highly integrated SWIR pushbroom camera.

Through the centralized architecture and chip-sharing strategy—for example, using a single FPGA to process data from all detectors and multiplexing driving and power supply signals—the volume and power consumption of the electronic subsystem were significantly reduced. As a result, the total system power consumption was maintained at approximately 12 W.

#### 2.2.2. Structural and Thermal Design Optimization

With sixteen InGaAs detectors (Zhongke Dexin Perception Technology Co., Ltd., Wuxi, China) uniformly distributed across a circular focal plane, the focal plane structural design must simultaneously satisfy the following requirements:

- ensuring that all detectors are precisely located on a common designed focal plane;
- providing efficient thermal conduction paths to maintain stable detector temperatures;
- accommodating spatial constraints under high-density detector arrangement.

During the packaging process of InGaAs detectors, assembly tolerances inevitably introduce variations in the distance between the photosensitive surface and the ceramic package base. Consequently, the actual focal surface position cannot be determined solely according to the theoretical back focal distance. In addition, the presence of detector windows limits the ability of coordinate measuring machines (CMMs) to directly measure the photosensitive surface. Therefore, adopting a traditional approach based on individual mechanical fine adjustment would significantly increase alignment complexity.

From a thermal perspective, InGaAs detectors employ ceramic packaging with an integrated thermoelectric cooler (TEC). Heat generated during device operation must be efficiently conducted through the package base to the silicon carbide (SiC) substrate to ensure long-term operational stability. As a result, the focal plane structure must simultaneously satisfy both optical precision and thermal conduction requirements.

**Figure 4:**
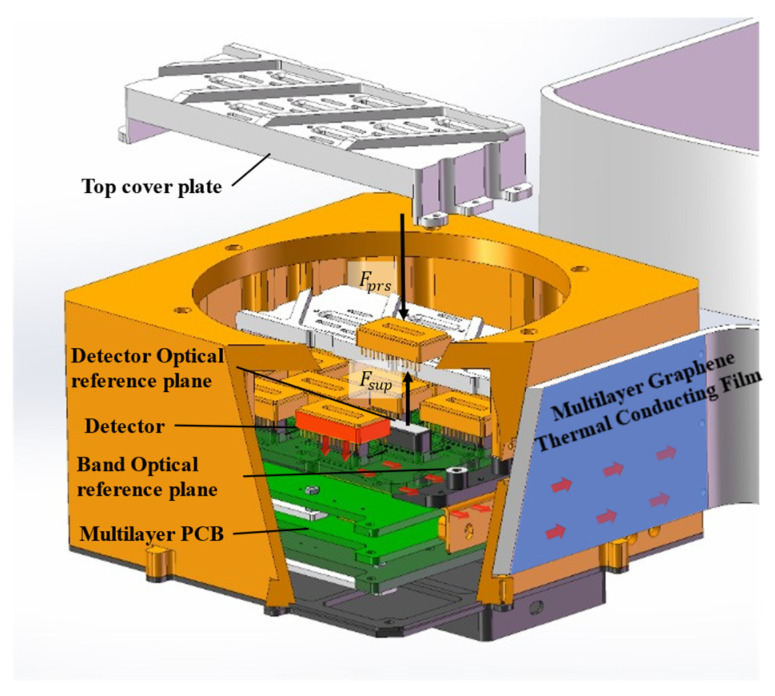
Detector stacked mounting structure and heat conduction path. The red arrows indicate the heat flow conduction path.

Under the constraint of limited lateral space, a sandwich-type stacked clamping structure was adopted (Figure 4). The upper layer consists of an aluminum alloy cover plate that applies uniform preload to the detectors. The middle layer is a circuit board that provides electrical connections and a certain degree of elastic buffering. The lower layer is a SiC substrate with sixteen independent protruding pads, serving as both the optical mounting reference and the primary thermal conduction interface.

Mounting posts are arranged on both sides of the SiC substrate to serve as reference points for coarse alignment of the entire focal plane assembly. The bottom surface of each detector directly contacts the corresponding SiC protrusion through openings in the circuit board, enabling precise positioning and an efficient heat conduction path.

It should be noted that the focal plane in this work is not a monolithic long-chip InGaAs array, but is composed of sixteen compact 1032 × 1 ceramic-packaged InGaAs linear detectors arranged in an interleaved mosaicking configuration. Each detector has a mechanical size of 28 mm × 15 mm × 3.18 mm and an active line length of 12.9 mm with a pixel pitch of 12.5 μm. Under active thermal control, thermal simulation shows that the temperature variation within a single detector is ≤0.2 °C, while the temperature difference among the photosensitive areas of different detectors is also ≤0.2 °C. Correspondingly, the measured pixel-to-pixel dark-current non-uniformity of a single detector is within 3%, indicating that the influence of thermal gradient on dark-current spatial non-uniformity is limited in the present design. To provide sufficient adjustment margin for focal plane leveling, each protrusion was intentionally manufactured with a small height allowance during the initial machining stage. After the initial system assembly, optical testing is performed to determine the actual defocus of each detector. The corresponding protrusions are then precisely machined to compensate for the measured deviations, allowing the focal plane to converge to the designed focal surface.

It should be noted that while this stacked structure significantly improves thermal management capability and overall mechanical stiffness, it also prevents independent mechanical focusing of individual detectors. Therefore, by exploiting the machinability of the SiC protrusions, a global focal plane leveling strategy based on imaging performance feedback must be established. This structural design thus provides the necessary foundation for the subsequent MTF-based focal plane leveling method.

### 2.3. MTF-Based Focal Plane Leveling Method for Multi-Detector Systems

#### 2.3.1. Relationship Between Defocus and MTF

The MTF is a fundamental metric for evaluating the spatial resolution performance of optical and optoelectronic imaging systems [22,23,24]. When the focal plane deviates from the ideal image plane, the system point spread function (PSF) broadens, leading to a noticeable attenuation of the system MTF, particularly in the mid- and high-spatial-frequency regions.

**Figure 5:**
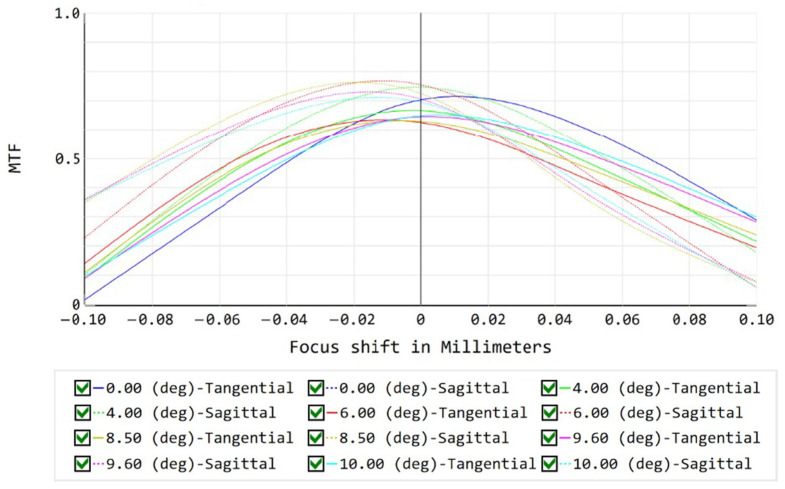
Simulated relationship between MTF and defocus amount. Simulated MTF as a function of defocus amount for different field angles, showing both tangential and sagittal responses.

Under near-axis conditions where the system is approximately diffraction-limited and optical aberrations are relatively small, the variation in MTF induced by defocus can be approximated as

(3) $$MTF(\nu ,\Delta z)\approx {MTF}_{0}(\nu )T(\nu ,\Delta z),$$

where ν denotes the spatial frequency, ${MTF}_{0}(\nu )$ represents the MTF in the absence of defocus, Δz is the equivalent image-plane defocus amount, and T(ν,Δz) is an additional attenuation factor introduced by defocus. For a given spatial frequency and optical system parameters (e.g., focal length, aperture, and F-number), the MTF reaches its maximum when Δz=0. As |Δz| increases, the MTF decreases monotonically. The Zemax OpticStudio (Ansys Zemax, Kirkland, WA, USA) simulation results for the proposed system, shown in Figure 5, confirm this trend.

Therefore, when other conditions remain unchanged, maximizing the MTF can be considered equivalent to minimizing the defocus amount. This relationship provides a direct performance-based quantitative criterion for focal plane leveling, rather than relying solely on structural dimensional tolerances.

It should be noted that this relationship depends only on the geometrical and wave-optical characteristics of the imaging system and is independent of the detector packaging structure. For imaging systems with different F-numbers or operating spectral bands, the corresponding MTF-Δz relationship can be obtained simply by selecting appropriate illumination sources and test targets.

#### 2.3.2. Mapping Between Object-Side Displacement and Image-Side Defocus

To quantitatively estimate the defocus amount, it is necessary to convert a controllable object-side axial displacement into an equivalent image-plane defocus.

**Figure 6:**
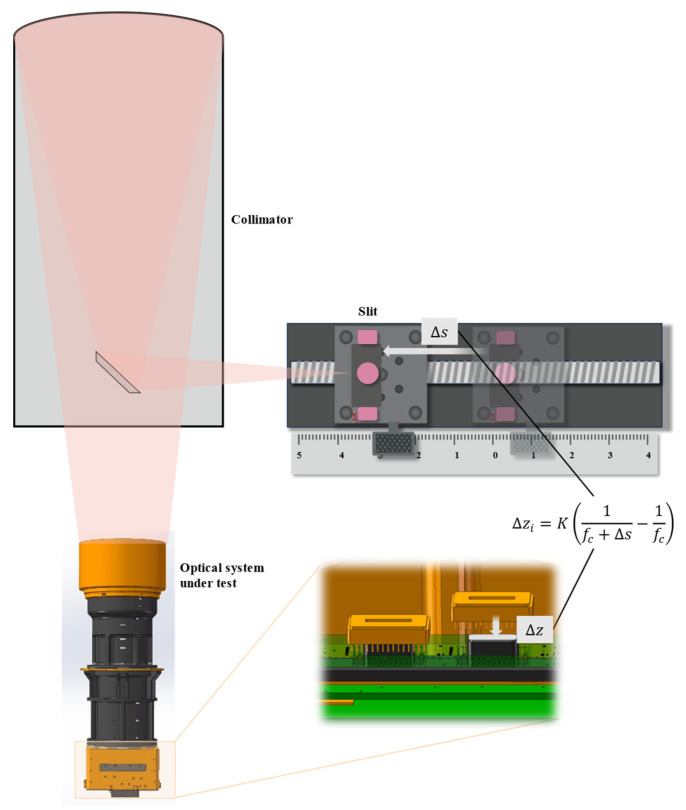
Schematic of the MTF measurement setup using a slit target. The pink shaded regions indicate the optical path. The overlapping semi-transparent shapes represent different positions of the slit target during the through-focus scan. Δs denotes the axial displacement of the slit target, and Δz denotes the equivalent image-plane defocus.

In this study, a measurement setup consisting of a collimator and a square-aperture slit target was employed (Figure 6). During the measurement process, the camera under test and the linear detector array were rigidly fixed as a single assembly. The test target was positioned near the focal plane of the collimator and translated precisely along the optical axis. The collimator is used to convert the light emitted from the slit target into a parallel beam, thereby simulating an object located at infinity for the camera under test.

The collimator converts small positional variations in the target plane into slight angular changes in the outgoing beam. When the target undergoes an axial displacement Δs near the object-side focal plane, a small angular perturbation Δθ is introduced in the emitted beam. After imaging by the tested optical system, this perturbation becomes equivalent to an image-plane defocus Δz. Under paraxial and small-angle approximations, the following relationship can be obtained:

(4) $$\Delta z\approx K\left(\frac{1}{f_{c}+\Delta s}-\frac{1}{f_{c}}\right),$$

where $f_{c}$ is the focal length of the collimator. The proportionality constant K is given by

(5) $$K=\frac{2f_{i}^{2}y}{D},$$

where $f_{i}$ is the focal length of the optical system under test, D is the aperture diameter of the system, and y denotes the width of the test target. The constant K depends only on the focal length and aperture of the optical system as well as the parameters of the collimator, and it can be obtained either through geometrical optics derivation or calibration experiments.

It is important to note that Δz is not directly coupled with pixel size or the specific operating spectral band. Therefore, the form of this mapping model remains unchanged for imaging systems with different focal lengths, F-numbers, or spectral bands; only the corresponding system constant needs to be updated.

#### 2.3.3. Closed-Loop Leveling Procedure Based on MTF

Based on the structural characteristics of the SiC focal plane substrate—featuring sixteen independent protruding pads—a closed-loop leveling procedure was established as follows:

1. Initial installation and coarse alignment.

During the initial machining stage, all protruding pads are manufactured with a small height allowance, so that the overall focal plane is intentionally positioned slightly toward the front-focus direction. A six-degree-of-freedom adjustment mechanism is then used to align the overall optical system.

2. MTF scanning and defocus estimation.

The target position is scanned along the optical axis, and the MTF curves of each detector are measured at selected spatial frequencies. The axial displacement corresponding to the peak MTF value, denoted as $∆s_{peak}$, is determined. The equivalent defocus amount Δz is subsequently calculated using the mapping model described above.

3. Calculation and machining of protrusion compensation.

The calculated defocus value is converted into the required height compensation ∆h for the corresponding SiC protrusion. Precision machining is then performed to adjust the heights of all sixteen protrusions in a single processing step.

4. Reassembly and verification testing.

After the detectors are reinstalled, MTF measurements are repeated. The improvement in MTF performance and the reduction in inter-detector variability are evaluated to verify the effectiveness of the leveling process.

This procedure achieves a transition from structure-based reference control to imaging-performance-based feedback control. By utilizing the machinable SiC protrusion structure, a closed-loop leveling mechanism—comprising measurement, compensation, and verification—is established. Since the model parameters depend only on the focal length, aperture of the imaging system, and the measurement setup, the method requires only recalibration of the proportional constant when applied to systems with different F-numbers or operating spectral bands.

Consequently, the proposed approach can be regarded as a performance-feedback-based focal plane leveling method, particularly suitable for compact and highly integrated multi-detector mosaicking imaging systems.

### 2.4. Optical Design

The optical system was developed for a short-wave infrared (SWIR) pushbroom remote sensing camera operating in the spectral range of 1000–2000 nm, with an effective focal length of 260 mm and an F-number of 2.6. To satisfy the stringent requirements of high radiometric sensitivity and broadband imaging performance, a large-aperture multi-element refractive configuration was adopted.

**Figure 7:**
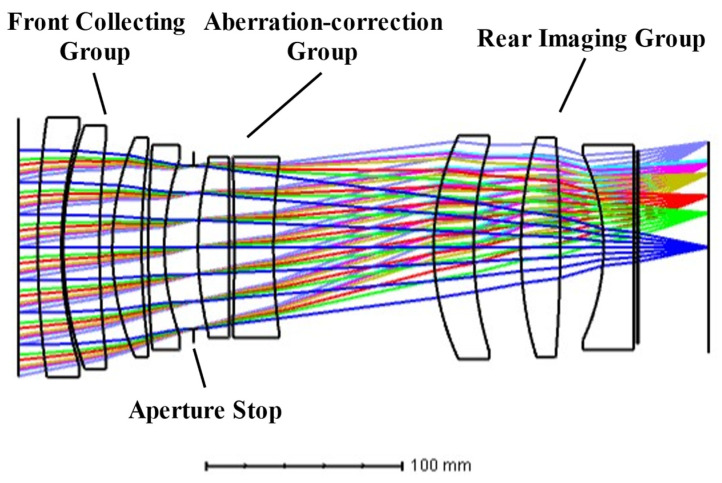
Optical layout of the SWIR imaging system. The colored lines represent ray paths at different field angles.

The optical layout employs a multi-group separated design, consisting of a front collecting group, an intermediate aberration-correction group, an aperture stop, and a rear imaging group, as illustrated in Figure 7. This configuration provides sufficient design freedom to simultaneously correct chromatic and monochromatic aberrations across the entire spectral band while maintaining high imaging performance under a large relative aperture condition.

In wide-swath pushbroom remote sensing systems, vignetting and non-uniform relative illumination directly affect radiometric consistency and image interpretability [25,26,27]. For the F/2.5 large-aperture SWIR optical system developed in this study, particular attention was devoted to vignetting suppression during the optical design stage. By adopting the multi-group separated optical architecture, carefully optimizing the aperture stop position, and coordinating the effective diameters of individual lenses, both mechanical vignetting and optical vignetting were effectively suppressed across the full field of view.

**Figure 8:**
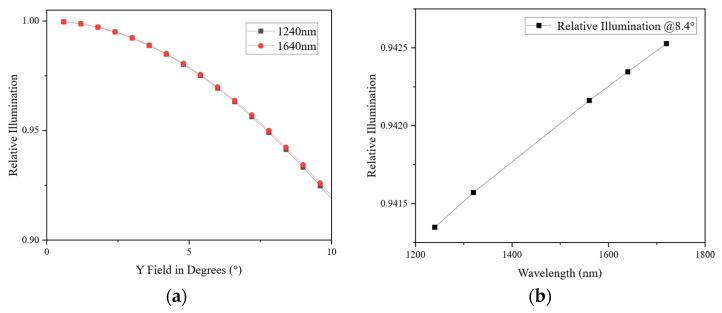
Simulation results of the full-field relative illumination distribution: (**a**) relative illumination versus field angle; (**b**) relative illumination versus wavelength.

The multi-element refractive configuration enables the gradual redistribution of ray bundles toward the image plane, preventing abrupt illumination loss at large field angles. Simulation results, shown in Figure 8a, indicate that within the 1000–2000 nm spectral range, the relative illumination exhibits a smooth and monotonic decrease from the center toward the edge of the field. The edge field maintains a relative illumination value exceeding 0.94, without any abrupt drop caused by mechanical obstruction or severe vignetting. This provides a solid optical foundation for subsequent radiometric calibration and maintaining a favorable signal-to-noise ratio (SNR) during on-orbit operation.

To suppress out-of-field stray light, a front baffle was introduced at the entrance of the optical system, and light-absorbing threaded structures together with anti-reflection/blackened surface treatment were applied to the inner walls of the baffle and lens barrel. In addition, the aperture stop helps limit the propagation of non-imaging rays within the system.

## 3. Results

### 3.1. Focal Plane Leveling Results

This section presents the experimental verification of the method described in Section 2.3. Using the MTF-based closed-loop leveling procedure, the defocus of sixteen InGaAs linear array detectors was measured individually, followed by SiC protrusion machining compensation and subsequent remeasurement. The measured MTF values before and after leveling, together with the corresponding defocus values and machining compensation amounts for each detector, are summarized in Table 3.

Overall, the results demonstrate that a single closed-loop leveling cycle significantly improves the imaging consistency across the multi-detector focal plane, which can be analyzed from the following aspects.

First, from the perspective of individual detectors, the majority of detectors exhibit a noticeable increase in MTF near the operating spatial frequency after leveling. For the 1240 nm channel, most detectors show an increase in MTF from approximately 0.2 in the initial assembly state to around 0.3 after leveling. In the 1640 nm channel, the improvement is even more pronounced for several detectors, with the maximum increase approaching 0.2. A small number of detectors exhibit minimal change before and after leveling; these correspond to detectors that were already close to the optimal focal plane in the initial assembly. This observation is consistent with the data in Table 3, where their measured defocus values and corresponding machining compensation amounts are close to zero.

Second, from the perspective of inter-detector consistency, significant variation in MTF values is observed among detectors prior to leveling, with some detectors clearly operating under a defocused condition. After applying the MTF-based quantitative defocus estimation and correcting the heights of the corresponding SiC protrusions, the MTF values of most detectors in both spectral channels converge to a relatively concentrated range, substantially reducing the variation among detectors. This convergence indicates that the focal plane as a whole has returned to a position close to the designed focal surface. The improvement directly reflects the advantage of the performance-driven leveling strategy, in which imaging performance serves as the primary evaluation criterion rather than relying solely on structural alignment references.

Furthermore, detectors exhibiting larger defocus values can be restored to a performance level comparable to the reference detectors through a single machining compensation step, without requiring repeated disassembly of the opto-mechanical system or iterative mechanical adjustments of individual detectors. This result demonstrates that by using the SiC substrate protrusions as machinable optical reference surfaces, combined with MTF-based feedback, it is possible to perform a one-time global correction of the multi-detector focal plane without introducing additional complex alignment mechanisms. Such an approach is particularly suitable for the compact and space-constrained mosaicked focal plane architecture employed in this work.

It should be noted that certain differences in MTF values among detectors still remain after leveling. On the one hand, the proposed method is fundamentally an indirect measurement approach based on MTF, which cannot directly provide the absolute deviation of each detector from the theoretical optimal focal plane. On the other hand, intrinsic variations in detector image quality, machining accuracy of the SiC protrusions, and residual errors in the opto-mechanical alignment process may also contribute to the final MTF differences. Therefore, the residual discrepancies shown in Table 3 can be interpreted as the combined manifestation of the overall system error budget at the focal plane level.

### 3.2. Spatial Resolution

The spatial resolving capability of an imaging system determines its ability to distinguish fine-scale ground features and is therefore a critical performance metric for quantitative remote sensing applications. The GSD describes the projected spacing between adjacent pixels on the ground, and its theoretical formulation for the present system has been discussed in detail in previous work [28]. However, in practical imaging systems, spatial resolution is influenced not only by pixel size but also by optical aberrations, detector response characteristics, and system-level alignment errors. Consequently, GSD alone cannot fully represent the effective resolving capability of the system [29].

To provide a more comprehensive evaluation of the actual imaging performance, the ground resolved distance (GRD) is introduced as an integrated performance metric. GRD represents the minimum separation between two adjacent ground targets that can be reliably distinguished in the image. It can be estimated from on-orbit imagery by measuring the FWHM of the LSF [29,30,31]. The LSF can be derived by differentiating the edge spread function (ESF) obtained from high-contrast edge targets [32].

Previous studies indicate that when the LSF FWHM lies within the range of 1.0–2.0 pixels, the system generally achieves a balanced imaging sharpness; values exceeding 2.0 pixels typically indicate significant image blurring [33].

**Figure 9:**
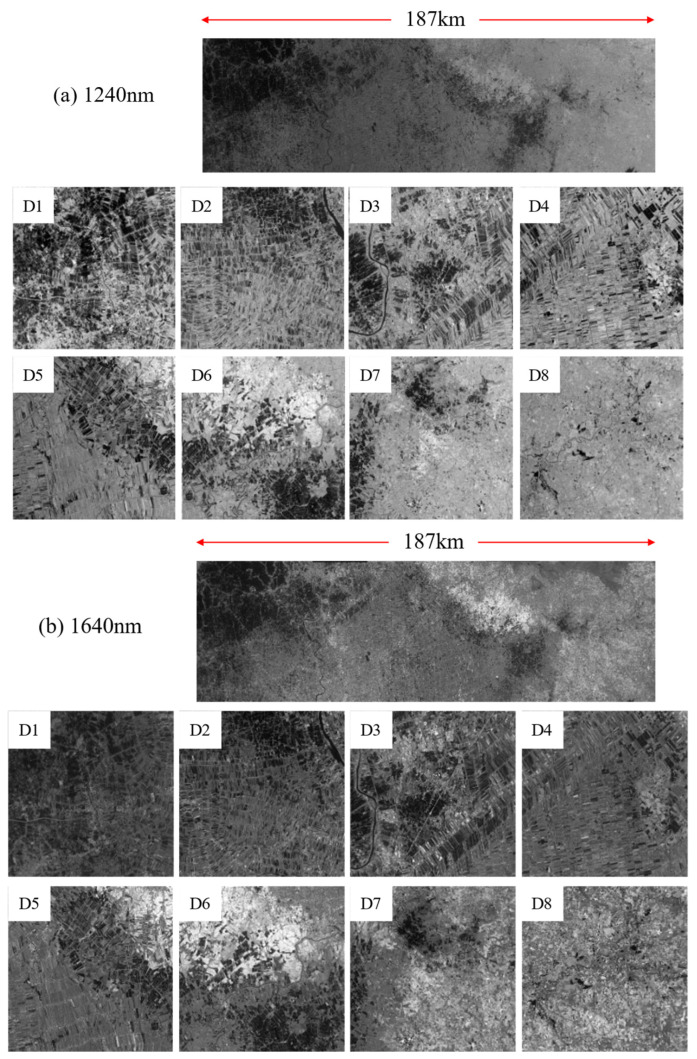
On-orbit imagery used for spatial resolution evaluation: (**a**) 1240 nm; (**b**) 1640 nm.

To verify the spatial resolution consistency after focal plane leveling of the multi-detector system, on-orbit imagery acquired over Bangkok, Thailand, on 21 December 2025, was selected for analysis (Figure 9). Within the eight imaging strips of the two spectral channels, natural targets with high contrast and well-defined edges—such as water–land boundaries and road edges—were selected. The ESF–LSF method was then applied to estimate the LSF FWHM for each strip, and the corresponding GRD values were subsequently calculated.

The nominal GSD of the instrument is 24 m, which is derived from the system design parameters reported in our previous work on the LHRSI instrument [28]. The evaluation results are summarized in Table 4. For the 1240 nm channel, the average LSF FWHM across the eight strips is 1.38 pixels, with a standard deviation of approximately 0.070 pixels, corresponding to an average GRD of 33.12 m. The 1640 nm channel exhibits similar characteristics, with an average LSF FWHM of 1.37 pixels and a strip-to-strip standard deviation of approximately 0.110 pixels, corresponding to an average GRD of 32.88 m.

Both spectral channels demonstrate low inter-strip variability, indicating good spatial resolution consistency across the mosaicked focal plane. This result is consistent with the convergence trend of detector MTF values discussed in Section 3.1, and it provides on-orbit validation of the effectiveness of the MTF-based focal plane leveling method in improving focal plane consistency.

It should be noted that the on-orbit spatial resolution metrics derived from the slanted-edge method represent system-level imaging performance. In the present study, no further decoupling was performed to separate the effects of atmospheric transfer and platform-motion-induced blur from the intrinsic opto-electronic response of the camera. Therefore, these on-orbit results are mainly used to evaluate the imaging consistency among mosaicked detectors and to verify the overall in-orbit system performance.

### 3.3. Signal-to-Noise Ratio

#### 3.3.1. SNR Stability Under Ground Thermal–Vacuum Conditions

Prior to launch, extensive ground-based experiments were conducted to verify the signal-to-noise ratio (SNR) performance of the infrared camera. The detailed testing methodology and baseline results have been systematically reported in previous work [17]. In addition to conventional performance tests, further verification was necessary because the system employs sixteen InGaAs detectors arranged in a high-density compact configuration. Although dedicated optimizations were implemented in both the electronic and structural design to stabilize the focal plane temperature and facilitate efficient heat dissipation, the noise characteristics of InGaAs detectors are known to be strongly correlated with operating temperature. Therefore, additional validation of the thermal control and heat dissipation strategy was carried out through thermal–vacuum experiments under high- and low-temperature conditions.

**Figure 10:**
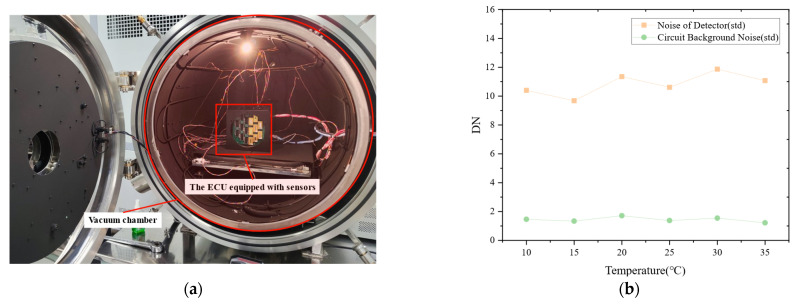
Ground-based thermal–vacuum verification of noise performance for the infrared camera: (**a**) experimental setup inside the thermal–vacuum chamber; (**b**) variation in detector noise and circuit background noise under different temperature conditions.

To simulate the thermal environment encountered during on-orbit operation, thermal–vacuum tests covering the expected temperature range were performed under ground conditions. The experimental setup and the corresponding noise variation results are presented in Figure 10.

The results indicate that the circuit background noise remains highly stable as the ambient temperature varies in the vacuum environment. Although the detector noise exhibits slight fluctuations with temperature changes, it remains generally stable at approximately 10 DN, without showing a significant increase at higher temperatures. Consequently, the overall system SNR remains at a consistently high level across the tested temperature range. These results confirm the effectiveness of the adopted thermal control and heat dissipation design, demonstrating that the system can effectively suppress temperature-induced noise drift.

#### 3.3.2. On-Orbit SNR Performance Under Multi-Scene Observations

After launch, the SNR performance of the system was further evaluated using actual on-orbit observation data. Because the payload is not equipped with an onboard radiometric calibration device, an approximate estimation approach based on homogeneous natural targets was adopted [34,35,36,37]. Specifically, regions with relatively uniform radiometric characteristics were selected, and the SNR was calculated in the radiance domain as the ratio of the mean Top-of-Atmosphere (TOA) radiance to its standard deviation within each selected region of interest (ROI). The conversion from raw digital numbers (DN) to TOA radiance was performed using the absolute radiometric calibration coefficients obtained from pre-launch laboratory calibration.

**Figure 11:**
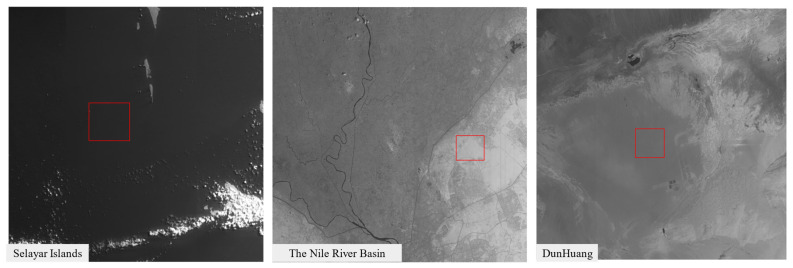
Representative homogeneous targets from different scenes and latitudes used for on-orbit SNR evaluation. The red boxes indicate the selected homogeneous regions of interest in each scene.

Representative scenes with different latitudes and radiometric properties were selected, including ocean surfaces, river basins, and desert/Gobi regions, as illustrated in Figure 11. The corresponding observation parameters and calculated SNR values are summarized in Table 5.

The results indicate that for low-reflectance targets, such as ocean surfaces, the SNR values of the 1240 nm and 1640 nm channels are approximately 66 and 48, respectively, demonstrating the system’s capability to detect relatively weak signals. In contrast, for high-reflectance scenes, such as desert and Gobi regions, the SNR values of both channels exceed 270, with peak values reaching approximately 340, indicating that the system maintains a high dynamic range and strong peak SNR performance under high radiometric input conditions.

The variation in SNR among different scenes mainly arises from differences in target reflectance and radiometric intensity, rather than instability in the intrinsic system noise performance. Overall, the results demonstrate that the system maintains stable noise characteristics under on-orbit conditions, enabling reliable signal discrimination across a wide range of radiometric input levels.

It should be noted that, because the dataset used in this study lacks synchronous ground-truth reference measurements, the estimated SNR values primarily reflect the stability of the system’s radiometric measurement performance, rather than providing a direct quantitative assessment of absolute radiometric accuracy.

## 4. Discussion

The proposed system integrates a multi-detector mosaicking architecture with a centralized ECU design, enabling lightweight and low-power implementation while maintaining a swath on the order of hundreds of kilometers and spatial resolution at the scale of several tens of meters. Compared with the conventional architecture in which each detector is driven by an independent electronics module, the centralized ECU configuration significantly reduces the electronic cost per imaging swath. As a result, the overall system mass and power consumption can be maintained within the limits acceptable for resource-constrained platforms. This demonstrates that, with appropriate functional partitioning and chip-sharing strategies, multi-detector mosaicked systems can still achieve a high level of system integration.

From the perspective of opto-mechanical and thermal performance, the SiC substrate combined with the stacked clamping structure provides a stable optical reference while simultaneously establishing an efficient thermal conduction path. Within the currently available ground-test and on-orbit datasets, no evident thermal-drift-related degradation in image quality has been observed, indicating that the system maintains good thermal and structural stability under the complex space environment. Although the on-orbit validation period remains relatively limited, the currently available data suggest that the proposed structural design can support stable system operation during the current mission phase.

Regarding the MTF-based focal plane leveling method, it should be noted that small deviations between the measured MTF values and the theoretical optimum remain for a few detectors after leveling. In this study, no secondary machining compensation was performed, primarily for the following reasons:

- After a single closed-loop compensation, most detectors already satisfy the imaging performance requirements of the system, and further improvement would provide only marginal gains in overall spatial resolution.
- Machining accuracy of the SiC protrusions, assembly tolerances, and measurement uncertainties collectively influence the measured MTF values; excessive compensation could introduce additional error sources.
- Under the constraints of limited resources and the need for rapid system deployment, a single closed-loop optimization provides a practical balance between engineering efficiency and performance improvement.

Therefore, the proposed method emphasizes performance-sufficiency-driven focal plane optimization, rather than pursuing theoretical extremum values. For mosaicked focal plane systems characterized by compact structures and limited capability for independent mechanical adjustment of individual detectors, the workflow of unified measurement, unified compensation, and unified verification enables global focal plane convergence with relatively low alignment complexity, demonstrating strong engineering applicability.

It should also be noted that the proposed method relies on highly stable test equipment and accurate MTF inversion procedures. For imaging systems dominated by optical aberrations or operating outside near-axis conditions, the monotonic relationship between defocus and MTF may no longer strictly hold, and additional error analysis based on the specific optical design would be required. Consequently, when extending this approach to other systems, the monotonicity assumption between defocus and MTF should be verified in advance.

Overall, the system design and focal plane leveling strategy proposed in this work demonstrate strong engineering feasibility for resource-constrained, wide-swath small satellite payloads, providing a practical reference for the implementation of multi-detector mosaicked imaging architectures.

While the present instrument configuration has demonstrated good performance for the intended mission objectives, its spectral design should also be interpreted within the context of system-level trade-offs. The selected SWIR bands should therefore be understood as a mission-oriented engineering trade-off rather than a fully application-customized narrowband design. For the intended use in atmospheric correction support and scene discrimination, the current band settings do not constitute a substantial limitation. However, for more specialized quantitative retrieval tasks that rely on narrow absorption features, such as moisture-related or cirrus-sensitive observations, narrower and more application-specific band definitions would be preferable in future instrument configurations.

## 5. Conclusions

This study addresses the demand for wide-swath, high-resolution SWIR imaging on resource-constrained spaceborne platforms through the design and on-orbit validation of a compact dual-channel short-wave infrared camera. Under operating conditions of approximately 187 km swath width and 24 m nominal spatial resolution, the total system mass is controlled at 10.62 kg, achieving a high level of system integration and efficient utilization of platform resources.

By adopting a multi-detector interleaved mosaicking architecture combined with a centralized ECU design, a compact optoelectronic integrated system was developed. To address the focal plane consistency issues introduced by detector mosaicking, a closed-loop focal plane leveling method using MTF as the core evaluation metric was established and implemented. Through quantitative correction of the SiC substrate protrusions, the focal surfaces of multiple detectors were brought into confocal convergence, resulting in a significant improvement in overall imaging performance.

Evaluation of the on-orbit imaging data indicates that both the spatial resolution and SNR meet the design specifications, confirming the effectiveness of the optical–mechanical–thermal design as well as the focal plane leveling strategy. These results demonstrate that, even under conditions of compact structure and limited platform resources, performance-driven focal plane compensation can effectively achieve engineering-level optimization of mosaicked multi-detector focal planes.

While maintaining the advantages of lightweight design and low resource consumption, the proposed camera successfully achieves wide-swath SWIR imaging capability, providing a practical engineering pathway for the miniaturization and constellation deployment of infrared payloads. Furthermore, the relatively independent and modular optical and electronic architectures offer favorable conditions for standardized payload integration in future missions.

## Figures and Tables

**Table 1:** Comparison of swath coverage and swath-to-mass efficiency among representative spaceborne SWIR imaging payloads.

System	Swath Width (km)	SWIR Spatial Resolution (m)	Mass (kg)	Volume	Power Consumption (W)	Swath-to-Mass Ratio (km/kg)	Mass per Swath per Band (kg/km·Band)
Landsat 8 OLI	185	30	450	Not announced	375	0.41	0.27
Sentinel-2 MSI	290	20	290	1 m^3^	250	1.00	0.076
On-orbit validated system (this work)	187	24	10.62	0.51 m × 0.18 m × 0.18 m	12	17.61	0.0265

The proposed work provides a scalable technical pathway for system-level trade-off analysis and engineering implementation of wide-swath, high-resolution SWIR push-broom imaging systems under resource-constrained platform conditions.

**Table 2:** Main specifications of the proposed SWIR push-broom camera.

Specification	Value
Imaging Mode	Push-broom imaging
Spectral Bands	Band 1: 1180–1300 nm Band 2: 1580–1700 nm
Orbital Altitude	500 km
Spatial Resolution (GSD)	24 m
Swath Width	187 km
F-number	2.6
Dimensions (mm)	514.7 × 180 × 180
Mass	10.62 kg

**Table 3:** Defocus values, SiC protrusion machining compensations, and MTF improvement results for all detectors.

1240 nm Channel						1640 nm Channel					
Detector	Initial MTF	$\mathrm{Target}\;\mathrm{Axial}\;\mathrm{Displacement}\;\Delta s_{i}$ (mm)	$\mathrm{Defocus}\;\Delta z_{i}$ (mm)	MTF After Compensation	MTF Improvement	Detector	Initial MTF	$\mathrm{Target}\;\mathrm{Axial}\;\mathrm{Displacement}\;\Delta s_{i}$ (mm)	$\mathrm{Defocus}\;\Delta z_{i}$ (mm)	MTF After Compensation	MTF Improvement
1240-D1	0.19	−30.30	0.1100	0.28	+0.09	1640-D1	0.25	−28.20	0.1050	0.37	+0.12
1240-D2	0.22	−30.30	0.1050	0.37	+0.15	1640-D2	0.11	−26.20	0.1040	0.34	+0.23
1240-D3	0.21	−19.20	0.0790	0.29	+0.08	1640-D3	0.12	−10.20	0.0610	0.23	+0.11
1240-D4	0.24	−20.20	0.0660	0.36	+0.12	1640-D4	0.22	−10.20	0.0160	0.38	+0.16
1240-D5	0.22	−12.20	0.0330	0.25	+0.03	1640-D5	0.21	−16.70	0.0550	0.30	+0.09
1240-D6	0.35	0.00	0.0000	0.33	−0.02	1640-D6	0.19	0.00	0.0000	0.23	+0.04
1240-D7	0.34	0.00	0.0000	0.30	−0.04	1640-D7	0.34	0.00	0.0000	0.35	+0.01
1240-D8	0.27	−33.40	0.1250	0.34	+0.07	1640-D8	0.27	−16.70	0.0730	0.32	+0.05

$∆s_{i}$ denotes the axial displacement of the slit target in the collimator test setup during the through-focus scan.$\;\Delta z_{i}$ represents the equivalent image-plane defocus obtained from $∆s_{i}$ using the mapping model described in Section 2.3.2.

**Table 4:** On-orbit LSF FWHM and corresponding GRD evaluation results.

Band	Mean LSF FWHM (Pixels)	Standard Deviation of LSF FWHM (Pixels)	Mean GRD (m)
1240 nm	1.38	0.070	33.12
1640 nm	1.37	0.110	32.88

**Table 5:** On-orbit SNR evaluation results under different scene and latitude conditions.

Imaging Region	Longitude, Latitude (°)	Solar Elevation Angle (°)	Acquisition Time	SNR (1240 nm)	SNR (1640 nm)
Selayar Islands	120.3903709°, −3.6196541°	64.16976	5 December 2025 10:21:22	66	48
The Nile River Basin	31.3708495°, 31.5167883°	62.08331	25 August 2025 16:37:58	338.8	346
Dunhuang	94.6421392°, 40.271072°	62.44299	1 August 2025 12:32:44	273.1	274

## Data Availability

The data that support the findings of this study are available from the corresponding authors.
